# Regional and local determinants of drought resilience in tropical forests

**DOI:** 10.1002/ece3.8943

**Published:** 2022-05-24

**Authors:** Renan Köpp Hollunder, Mário Luís Garbin, Fabio Rubio Scarano, Pierre Mariotte

**Affiliations:** ^1^ Programa de Pós‐graduação em Ecologia IB, CCS, Ilha do Fundão Universidade Federal do Rio de Janeiro Rio de Janeiro Brazil; ^2^ Departamento de Biologia Centro de Ciências Exatas, Naturais e da Saúde Alto Universitário Universidade Federal do Espírito Santo Alegre Brazil; ^3^ Agroscope Grazing Systems Nyon Switzerland

**Keywords:** climate change, El Niño, growth, mortality, recovery, resistance

## Abstract

The increase in severity of droughts associated with greater mortality and reduced vegetation growth is one of the main threats to tropical forests. Drought resilience of tropical forests is affected by multiple biotic and abiotic factors varying at different scales. Identifying those factors can help understanding the resilience to ongoing and future climate change. Altitude leads to high climate variation and to different forest formations, principally moist or dry tropical forests with contrasted vegetation structure. Each tropical forest can show distinct responses to droughts. Locally, topography is also a key factor controlling biotic and abiotic factors related to drought resilience in each forest type. Here, we show that topography has key roles controlling biotic and abiotic factors in each forest type. The most important abiotic factors are soil nutrients, water availability, and microclimate. The most important biotic factors are leaf economic and hydraulic plant traits, and vegetation structure. Both dry tropical forests and ridges (steeper and drier habitats) are more sensitive to droughts than moist tropical forest and valleys (flatter and wetter habitats). The higher mortality in ridges suggests that conservative traits are not sufficient to protect plants from drought in drier steeper habitats. Our synthesis highlights that altitude and topography gradients are essential to understand mechanisms of tropical forest's resilience to future drought events. We described important factors related to drought resilience, however, many important knowledge gaps remain. Filling those gaps will help improve future practices and studies about mitigation capacity, conservation, and restoration of tropical ecosystems.

## Graphical abstract

Drought resilience of tropical forests is affected by multiple biotic and abiotic factors varying at different scales. Here, we show that topography has key roles in controlling biotic and abiotic factors in each forest type. Our synthesis highlights that gradients of altitude and topography are essential to understand tropical forest's resilience to future drought events.

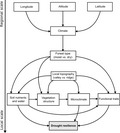

## 1 INTRODUCTION

El Niño drought is a natural phenomenon that strongly affects plants at different scales, from individuals to populations and communities, and thus ecosystems worldwide (Dai, 2011; Fauset et al., 2012). Drought events are predicted to modify species composition and abundance, as well as ecosystem functioning and dynamics (Boeck et al., 2017), especially in tropical forests (Reichstein et al., 2013) due to high exposure to El Niño events (IPCC, 2022). Nevertheless, resilience to droughts in tropical forests remains poorly understood (Meir et al., 2015; Xu et al., 2013). Identifying biotic and abiotic factors related to mortality and low growth induced by droughts will allow us to better understand and predict forest resilience and dynamics.

**FIGURE 1:**
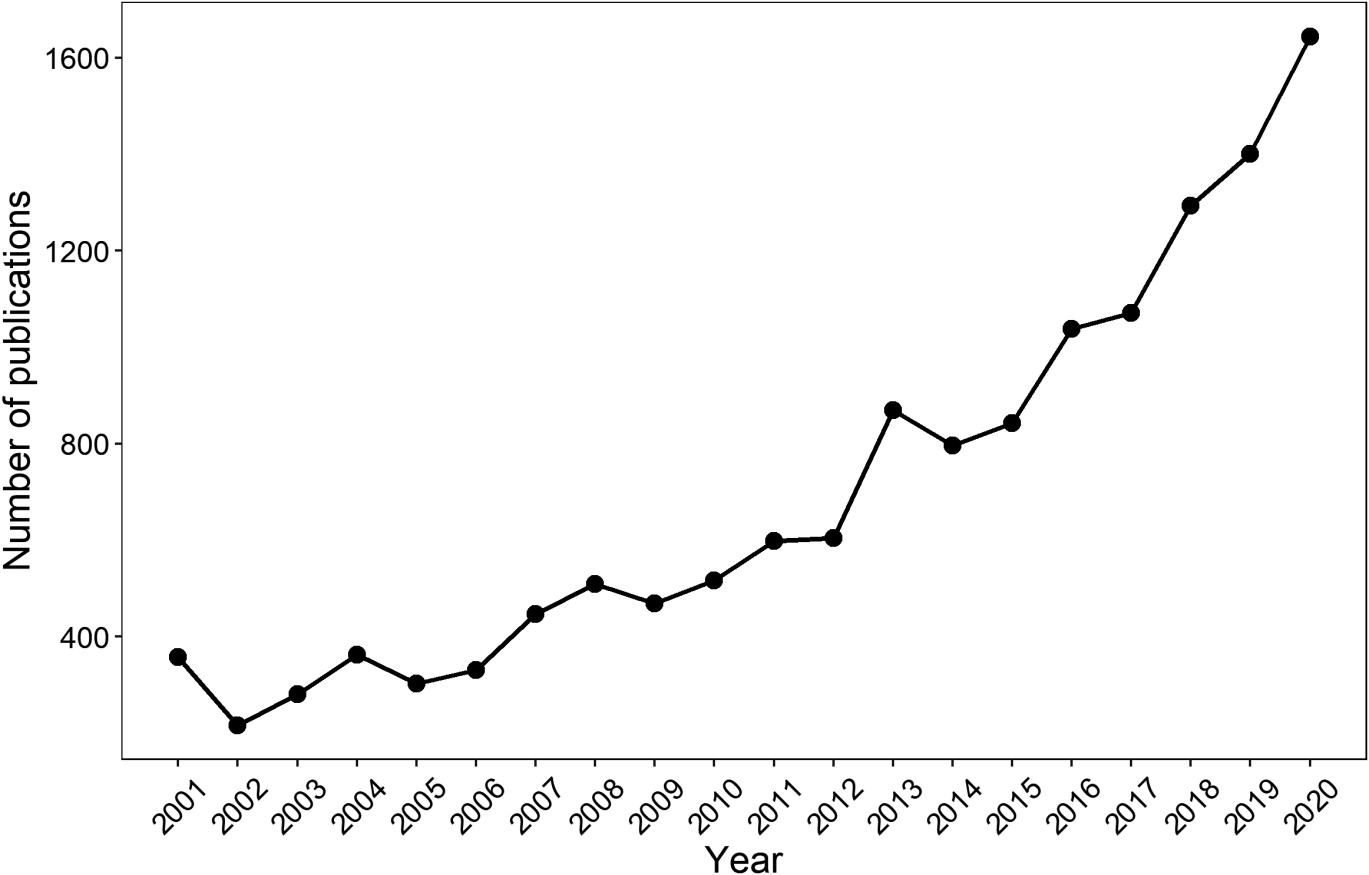
Number of publications related to drought effects in tropical forests during the last 20 years (search results in sciencedirect.com for keywords “drought” and “tropical forest”)

The interest in studying drought effects in tropical forests has increased more than four times during the last 20 years (Figure 1). Tropical forests were strongly affected by the two most severe droughts ever recorded in 1998/1999 (Slik, 2004) and 2015/2016 (Kogan & Guo, 2017; Otto et al., 2015). The most recent one led to a high net carbon loss, even higher than the 2010 drought in the Amazonian forest (Liu et al., 2017). Tropical forests provide key ecosystem services – such as water supply, carbon sequestration, pollination, and climate control – to urban and rural areas (Joly et al., 2014; Silva et al., 2021). Although our understanding of drought effects worldwide is growing, the impacts of droughts in tropical forests are still poorly understood, which limits our ability to model forest responses to future climate scenarios.

Repeated occurrences of strong droughts can select for drought‐tolerant species due to higher mortality of vulnerable species (Aguirre‐Gutiérrez et al., 2020). Thus, it has been predicted that trees of tropical forests will become smaller with denser wood (Phillips et al., 2010) due to high mortality rates of canopy trees and shade‐tolerant species (Fauset et al., 2012). There is a field of evidence that tropical forests can change to a drier ecosystem due to the interactions between recurrent fires and climate change. This process drives communities through a savannization (Sansevero et al., 2020) and/or a secondarization process (Barlow & Peres, 2008), thus decreasing carbon sequestration and enhancing climate change effects because dry ecosystems sequester less CO_2_ than moist forests (Taylor et al., 2017). Therefore, repeated drought events associated with high deforestation and recovery can drive tropical forests into a different type of forest following the future climate scenarios. This process can lead to changes in forest composition, vegetation structure, traits, and ecosystem services (Aguirre‐Gutiérrez et al., 2020). Predictions of which plant species are most vulnerable to drought effects and, where they occur, are needed to model consequences of climate change and to determine the best mitigation strategies.

Climate changes impact forests at multiple spatial scales, and this is the interaction of processes across scales that may determine forest resilience (Reyer et al., 2015). For instance, drought effects are influenced by gradients varying at global, regional, and local scales (O’Brien et al., 2017). Thus, looking at multiple spatial scales may offer the best way to predict forest resilience because plant mortality and reduced growth can occur locally with potentially negative effects, but they may smooth out at larger spatial scales (Reyer et al., 2015). The aim of this study was to synthesize biotic and abiotic factors, mostly controlled by topography that can be used to predict drought‐induced mortality in tropical forests. We expect that this review will help identify knowledge gaps in drought resilience research and provide a framework for future studies.

## 2 WHAT IS DROUGHT RESILIENCE AND HOW DO WE QUANTIFY IT?

In this review, we considered the ecological drought definition that has recently been proposed by Crausbay et al. (2017). An ecological drought is an episodic deficit in water availability that drives ecosystems beyond thresholds of vulnerability, impacts ecosystem services, and triggers feedbacks in natural and human systems (Crausbay et al., 2017). However, an El‐Niño drought also increases temperatures in tropical regions leading to higher vapor pressure deficit (or VPD; Grossiord et al., 2020). Many drought indices have been used to define and describe droughts in terms of severity, duration, and spatial extent (Mishra & Singh, 2010; Mukherjee et al., 2018). For instance, using the Standardized Precipitation Evapotranspiration Index (SPEI) that takes into account both precipitation and temperature will better describe El‐Niño droughts (Slette et al., 2019). SPEI values are grouped in the following categories: extremely dry (SPEI ≤ −2), severely dry (−2 < SPEI ≤ −1.5), moderately dry (−1.5 < SPEI ≤ −1), and near normal conditions (−1 < SPEI < +1). More negative SPEI values mean higher temperatures and lower precipitation, and are highly associated with mortality events (Greenwood et al., 2017). However, despite standardization of drought metrics has received much less attention, they are essential to describe the severity of droughts and to understand drought resilience of ecological systems (Van Meerbeek et al., 2021).

Resilience is the persistence of systems and their ability to absorb change and disturbance; still, they should maintain the same relationships between populations or state variables (Neubert & Caswell, 1997). Resistance is the ability of a system to maintain its properties and functions during a disturbance (Neubert & Caswell, 1997). Recovery is the similarity between the new condition, after a perturbation, and the conditions before the disturbance (Van Meerbeek et al., 2021; Westman, 1978). Thus, resilience can be assessed by measuring both resistance and recovery (Oliver et al., 2015). Droughts are disturbances associated with vegetation mortality and reduced growth (Adams et al., 2009; Greenwood et al., 2017; Meir et al., 2018; Verbesselt et al., 2016). Therefore, considering the detrimental impacts of droughts, quantifying mortality, and growth to estimate drought resilience is an important approach to measure vulnerability and drought resilience of plant species (Redmond et al., 2018). Evidences suggest a growth–survival trade‐off, showing, for example, that survival rates are negatively correlated with growth rates (O'Brien et al., 2017; Wright et al., 2010). Another pattern can be found when recovery rates are measured after a normal condition, for example, after a La Niña event. In this case, a resistance–recovery trade‐off is expected, with sites or species showing greater resistance rate during drought but lower recovery rate after a rainy period (Gazol et al., 2017). Furthermore, during the recovery period, new individuals can be recruited, mainly because of the available niches resulting from large tree mortality (Redmond et al., 2018). Tree resistance and recovery to droughts will likely determine the long‐term trajectory of tropical forests. Therefore, resilience is an important component to determine community and ecosystem sensitivity to disturbances and to predict shifts in forest carbon stocks caused by climate changes (Sánchez‐Salguero et al., 2018). Furthermore, low resilience is related to future mortality risk in trees (DeSoto et al., 2020) and repeated extreme climate events can exceeds the capacity of organisms or ecosystems to recover (Hoover et al., 2021).

Drought timing and severity, as well as forest type, are important factors to take into account when estimating the resistance and necessary time for the recovery. For instance, in 2017, savannas and grasslands had already recovered from the 2015/2016 drought but tropical forests did not because tropical trees have slower growth and recruitment rates (Wigneron et al., 2020). Drought legacy, i.e., the time of recovery after a drought, can take longer according to the drought magnitude (Huang et al., 2018; Kannenberg et al., 2020). For instance, tropical forests in Costa Rica recovered from the 1997 drought within 2 years (Silva et al., 2013) and the Puerto Rican forests recovered from the 2015 drought within 1 year (Schwartz et al., 2019, 2020). Quantifying the local magnitude of droughts can be difficult due to spatial variation in precipitation and temperature. An El Niño event does not mean the same dry condition because some forests can be more exposed to such conditions depending on the latitude and the topography.

## 3 DROUGHT RESILIENCE IN DIFFERENT TYPES OF FORESTS

**FIGURE 2:**
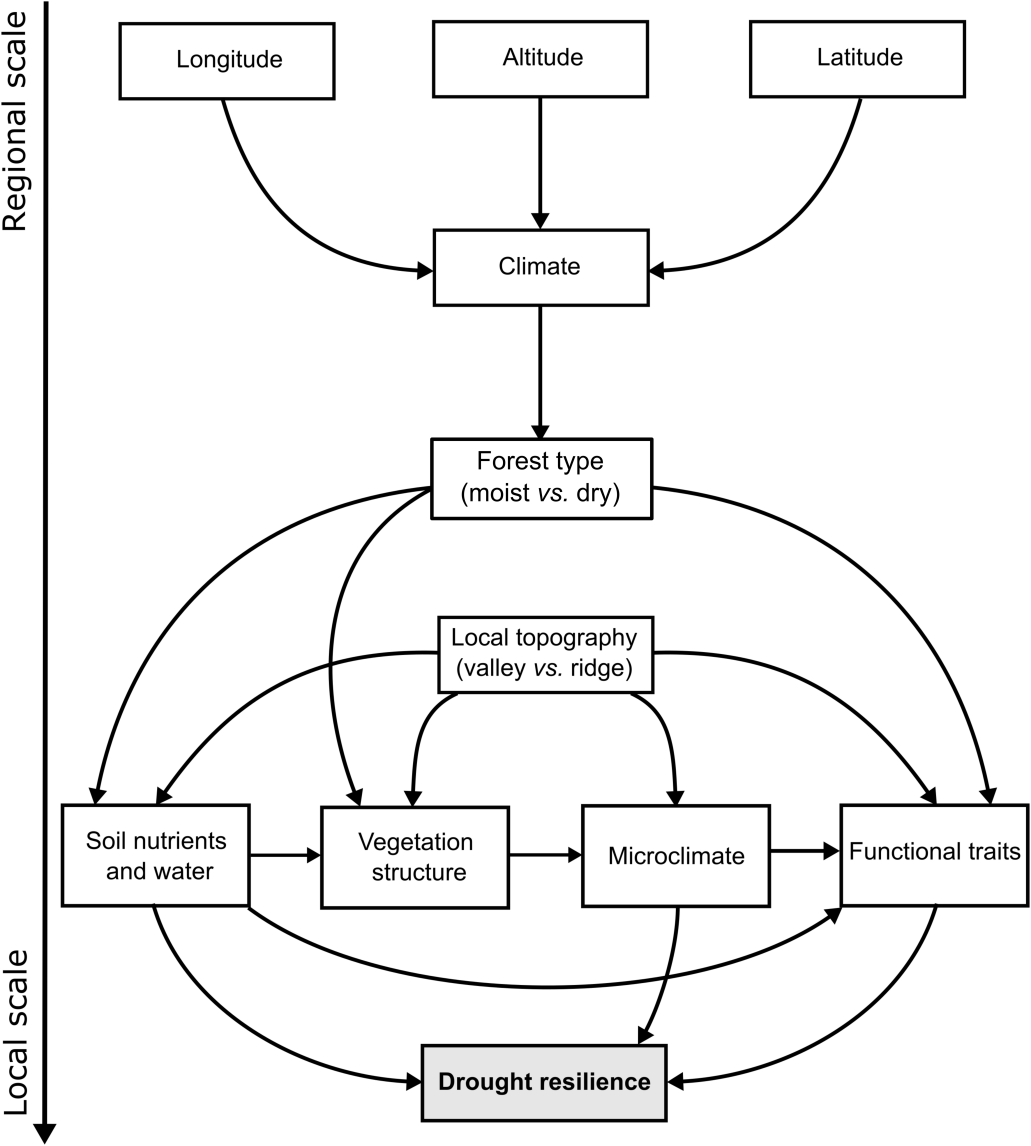
Conceptual model of biotic and abiotic factors mediating drought resilience in tropical forests. Topography is an important factor controlling abiotic and biotic factors related to drought resilience. Altitude, latitude, and longitude lead to high climate variation and to different forest types: moist and dry forests. Locally, topography is a key factor controlling biotic and abiotic factors related to drought resilience within each type of forest

Tropical forests include dry and moist forests (Dexter et al., 2018; Olson et al., 2001). Several other forest types have been identified (e.g., semideciduous forests, cerradão), but they are not clearly defined and are difficult to classify (Dexter et al., 2018). Semideciduous forests do not form a distinct type of forest, but they are found, instead, in moist forest sites (Dexter et al., 2018). Thus, we focused on the general classifications of dry and moist tropical forests. Dry tropical forests (also called seasonally dry tropical forest; *sensu* Pennington et al., 2000) range from tall forest on moister regions to shrublands on the driest regions and the vegetation is mostly deciduous during the dry season (Pennington et al., 2000, 2009). Moist tropical forests are characterized not only by the presence of evergreen plant species in high‐altitude moister areas of the tropics but also by the presence of deciduous species in areas with seasonal climatic regime (WCMC, 1992). Most species from dry forests tend to lose their leaves during the dry season impacting nutrient cycling and water availability (Reich & Borchert, 1984). Thus, these two types of forests are marked by differences in species composition and structure (Figure 2), as well as ecosystem processes (Dexter et al., 2018; Esquivel‐Muelbert et al., 2017). Furthermore, there are differences in soil fertility between these two forest types (Figure 2), with dry forests occurring on more fertile soils (Dexter et al., 2018; Pennington et al., 2000; Raulino et al., 2020). Therefore, forest classification (dry vs. moist tropical forest) is mainly driven by regional components, such as altitude, latitude, and longitude, that lead to differences in regional climate (precipitation and temperature) (Figure 2). Among these components, precipitation gradient is the main factor controlling the type of forest (Dexter et al., 2018).

Distinct patterns of drought resilience likely exist among different forest types because each type of forest will impact differently soil nutrients and water availability, vegetation structure, and functional traits (Figure 2). For instance, African tropical forests are more resistant to climatic extremes than Amazonian and Asian forests due to differences in their vegetation structure (Bennett et al., 2021). Furthermore, El Niño impacts vary latitudinally in the Atlantic Forest, with tropical areas being more impacted by El Niño events than subtropical areas (Rodrigues et al., 2011). Eastern and southern regions of the Amazon Forest experienced stronger drought impacts than the northwestern region (Van Schaik et al., 2018).

Tropical dry forests are generally less resistant to droughts (Allen et al., 2017; Oliveira et al., 2021). Furthermore, tropical forest communities in West Africa that normally experience higher seasonal water deficit, and that became drier through time, tended to become more homogeneous in functional, taxonomic, and phylogenetic diversity (Aguirre‐Gutiérrez et al., 2020). Some areas of tropical forest are secondary forest patches recovering from land use, timber extraction, or natural disturbances (Rüger et al., 2020). Pioneer species occurring in those secondary forests present high mortality during drought periods (Rocha et al., 2020), but high recovery rates after a drought event (Gazol et al., 2017; Poorter et al., 2016). Those evidences may suggest that the type of forest is an important factor controlling drought resilience because dry and wet forests distinguish in vegetation structure and soil nutrients (Dexter et al., 2018; Raulino et al., 2020; Figure 2), both important components affecting tree growth and mortality (Gessler et al., 2017; Hollunder et al., 2021).

## 4 DROUGHT RESILIENCE ACROSS TOPOGRAPHIC GRADIENTS

**FIGURE 3:**
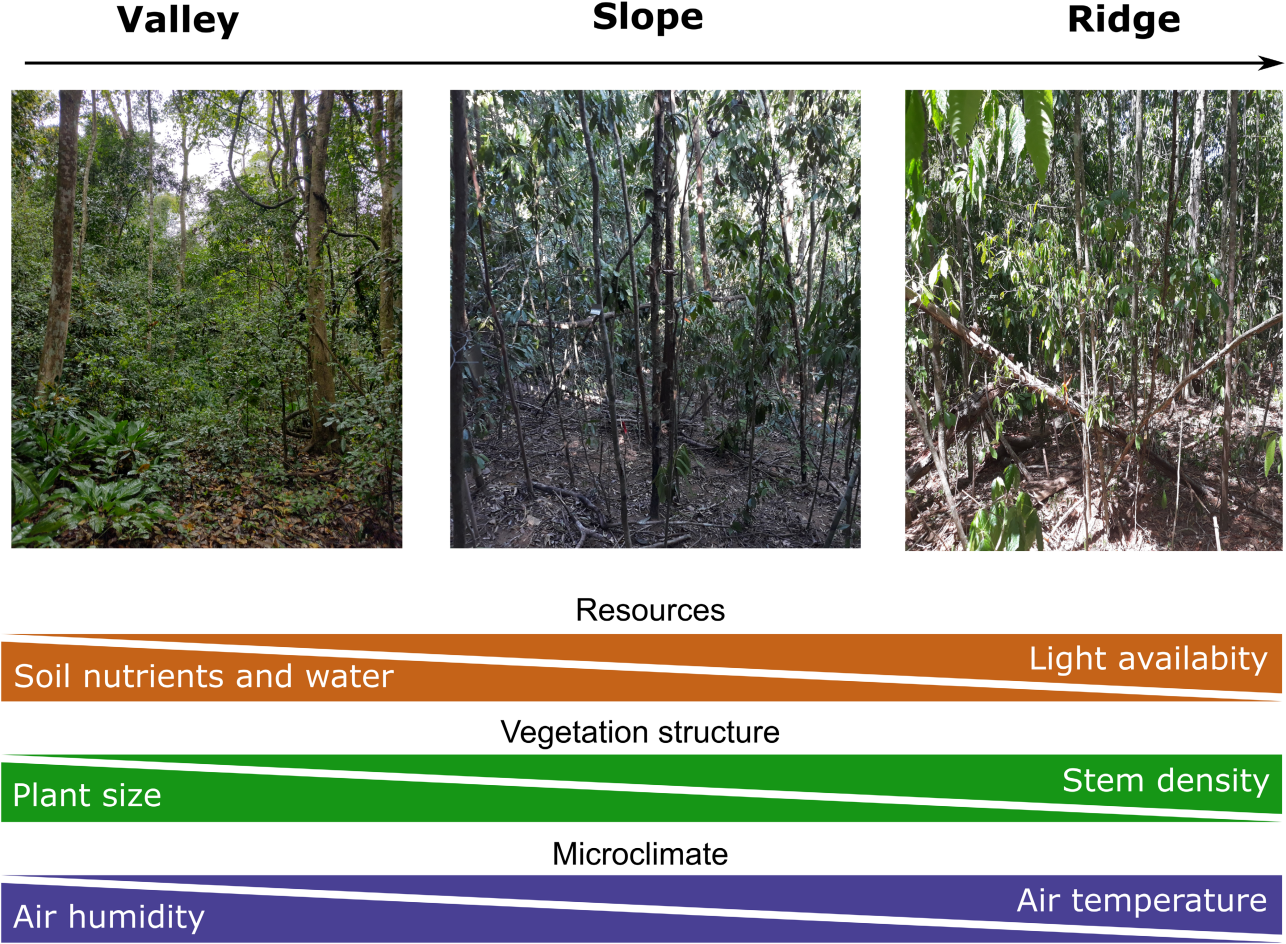
Schematic overview of the biotic (vegetation structure) and abiotic (resources and microclimate under the canopy) factors controlled by local topography in tropical forests. Valleys have higher soil nutrients and water availability, air humidity, and bigger trees in terms of DBH, height, and canopy cover. In turn, ridges have higher light availability, stem density, and air temperature. Slopes usually have intermediate values depending on the orientation and slope degree (low slope or high slope). The photos were taken in Mata das Flores State Park, Southeast Brazil

Locally, topography is also a source of environmental heterogeneity within each forest type (Jucker, Bongalov, et al., 2018; Nettesheim et al., 2018). Abiotic factors strongly vary across topographic gradients, such as water availability, soil nutrients, and microclimate (Figures 2 and 3, Fyllas et al., 2017). Topographic variation in tropical forests can be divided into three main habitats: valleys (flatter, wetter, and more fertile habitats), ridges (drier habitats and less fertile habitats), and slopes (steeper and drier habitats). Biotic factors, such as species traits and vegetation structure, are also driven by topography (Figures 2 and 3; Jucker, Bongalov, et al., 2018; Rodrigues et al., 2019). Topography is thus an important factor to take into account to understand drought impacts in plant communities due to the role of those factors in survival and growth of plant species (Hollunder et al., 2021; O'Brien et al., 2017).

Species occurring in different topographical habitats can show distinct patterns of mortality and growth rate because such species are under different environmental conditions. Most of the studies in tropical forests have shown that woody plant species occurring in valleys have lower mortality than the ones occurring in ridges and slopes during extreme droughts, such as during the 1998/1998 and 2015/2016 droughts (Table 1). Valleys can act as refuges, providing nutrients and water that protect plant species during droughts (Costa et al., 2020; Hollunder et al., 2021). Furthermore, shade availability (Holmgren et al., 2012) and shallow water table (Costa et al., 2022) in valleys can reduce drought impacts and improve the performance of plant species during drought periods. Species occurring in dry habitats are living under their microclimate limits and under water stress, and a small change in precipitation and temperature can be physiologically stressful (Aguirre‐Gutiérrez et al., 2020; Allen et al., 2017; Cartwright et al., 2020). Species from dry habitats can show high recovery rates during a post‐drought period (Schwartz et al., 2019), following the resistance–recovery trade‐off. However, this trade‐off is not always evident when dry habitats exhibit drought legacy effects due to impacts of extreme droughts (Allen et al., 2017; Anderegg, Schwalm, et al., 2015). The effects of drought in naturally drier habitats can be so significant that their effects on growth and mortality can last for years, despite the drought being mitigated meteorologically or hydrologically (Kannenberg et al., 2020).

**TABLE 1:** Studies that identified significant differences in tree mortality across topographical gradients during drought and non‐drought periods in tropical forests

References	Local topography			Location	Drought period
	Valley	Slope	Ridge		
Costa et al. (2020)	More resistant	–	Less resistant	Brazilian Amazon	2001–03; 2003–05; 2007–09; 2014
Hollunder et al. (2021)	More resistant	Less resistant	Less resistant	Brazilian Atlantic Forest	2014–2016
Nakagawa et al. (2000)	More resistant	More resistant	Less resistant	Malaysia	1997–1998
Schwartz et al. (2019)	More resistant	Less resistant	Less resistant	Puerto Rico	2015
Schwartz et al. (2020)	More resistant	Less resistant	–	Puerto Rico	2015
Esteban et al. (2021)	More resistant	Less resistant	Less resistant	Brazilian Amazon	2010/2015–2016
Itoh et al. (2012)	Less resistant	More resistant	More resistant	Malaysian Borneo	1997–1998
Zuleta et al. (2017)	Less resistant	More resistant	More resistant	Colombian Amazon	2010
Ferry et al. (2010)	More resistant	Less resistant	Less resistant	French Guiana	1991 – 2003
Toledo et al. (2011)	Less resistant	Less resistant	More resistant	Brazilian Amazon	2003–2008
Comita and Engelbrecht (2009)		More resistant	Less resistant	Barro Colorado	2002–2003

Note

Most of the studies have found that woody plants occurring in valleys are more resistant than those from the ridges and slopes. We considered bottomland as synonyms of valleys and plateaus as synonyms of ridges. Search results in webofscience.com for keywords: drought and mortality and “tropical forest” and topograph*.

Other studies have shown that valleys can show higher mortality than the ones occurring in ridges (Table 1). During a non‐drought period or moderate extreme drought, species from valleys and low slopes tend to have higher mortality rates comparing to species from ridges and slopes (Toledo et al., 2011; Zuleta et al., 2017). Species from valleys do not have traits related to drought tolerance, while species occurring in ridges have traits related to survival during periods of water stress (Allen et al., 2017; Gessler et al., 2017). Furthermore, valleys with higher groundwater table depth can exacerbate the dry period and increase tree mortality when compared to valleys with shallow groundwater table (Costa et al., 2022). Thus, if the valley does not provide water, nutrients, and a suitable microclimate, the plant mortality can increase during a severe drought. Such distinct evidences indicate that there is no general pattern of drought impacts for all tropical forests, although most of the studies found stronger resistance in wetter habitats. Biotic and abiotic factors controlled by topographic gradients are also strong drivers of tree growth and mortality, as well as of forest resilience, and can thus contribute to help elucidating such patterns (Figure 2).

## 5 ABIOTIC FACTORS

### 5.1 Water availability and soil nutrients

Climate changes are strongly related to changes in rainfall patterns worldwide, involving reductions in the total amount of annual precipitation and/or increase in the dry season duration (Grossiord et al., 2016). Furthermore, at local scales, soil water availability is highly variable along topographic gradients, with wetter valleys and drier ridges (Figure 3, Gibbons & Newbery, 2002). Both changes in soil moisture and reduced precipitation strongly drive changes in physiological traits (Pezzola et al., 2017). For instance, drought‐induced water stress decreases photosynthesis rate, which leads to slow and reduced plant growth (McDowell et al., 2008). In addition, low water availability decreases the physiological mechanisms (secondary metabolites) related to plant defense and favors the reproduction of insects (Mattson & Haack, 1987; McDowell et al., 2011), thus increasing woody species vulnerability to death (Das et al., 2016). Water availability also plays a vital role in forest recovery, increasing the growth rate of plant tissues during post‐drought periods (Álvarez‐Yépiz et al., 2018; Poorter et al., 2016). Water availability is an important driver of resistance and recovery rates due to its strong spatial variations caused by topographical gradients (Gibbons & Newbery, 2002) and temporal variations caused by El‐Niño events (Moura et al., 2019).

Soil nutrients also play an essential role in plant communities distributed across topographic gradients (Figure 3, Guan et al., 2015). Soil nutrients are key factors of photosynthesis, which is an essential mechanism of plant survival and growth (Fatichi et al., 2014). Nutrient stoichiometry can change across topographic gradients due to the unidirectional fluxes from ridges to valleys and of the consequent loss of nutrients in ridges (Werner & Homeier, 2015). Therefore, nutrient limitation can lead to high nutrient competition in ridges (Werner & Homeier, 2015). Nitrogen (N), phosphorus (P), and carbon (C) are the main elements required to plant growth and survival (Zhang et al., 2012, 2017), and change in their ratios, caused by droughts, can have strong consequences for the resilience of tropical forests (Gessler et al., 2017). For instance, P of tropical forest soil is positively correlated with growth rate and negatively related with mortality rate (Soong et al., 2020). Tropical plants in valleys may require larger amounts of leaf N to deal with the intense shading of forest understory (Torres‐Leite et al., 2019). Droughts can alter N and P cycles in ecosystems, either directly through changes in N mineralization and P sorption (Mariotte et al., 2017) or indirectly through changes in plant nutrient uptake and growth (Mariotte et al., 2020). Changes in litter N, P, and C content alter decomposition rates because decomposers require nutrients from either litter or soil for their functioning (Gartner & Cardon, 2004). Deficiency in plant‐available nutrients in ridges is enhanced through a positive feedback driven by poor litter decomposability (Werner & Homeier, 2015). Furthermore, more frequent droughts slow down litter decomposition and reduce plant‐available nutrients (da Silva et al., 2020), both affecting drought resilience (Gessler et al., 2017).

### 5.2 Microclimate variation across topographic gradients

Vegetation structure (e.g., height, stem density, and canopy biomass) and local topography (e.g., elevation, slope, and aspect) strongly influence the microclimate (Hardwick et al., 2015). Understory and canopy species are under different microclimatic conditions due to the vertical structuration in tropical forests. For instance, canopy cover of larger trees regulates the irradiance and light intensity entering the understory layer (Nepstad et al., 2002; Wright et al., 2010). Thus, the light availability is more variable and limiting for understory species than for large trees (Wright et al., 2010). The high drought‐induced mortality of large trees can change the microclimate of the understory (Redmond et al., 2018; Zellweger et al., 2020). Small forest fragments have lower abundance of large trees due to high edge effects (Dantas de Paula et al., 2011) and lack suitable microclimatic sites for the persistence of species during drought periods (Hardwick et al., 2015; Laurance et al., 2018). During a water stress period, the low leaf area of large deciduous trees leads to high light availability in the understory (Smith et al., 2019). In turn, the increase in light availability induces high rates of photosynthesis in the understory, thus affecting species adapted to shaded conditions (Guan et al., 2015).

Other microclimate variables, such as air temperature and humidity, are controlled by light availability that reaches the understory stratum. For example, in the valleys with dense forest canopy, air humidity is higher and temperature is lower than ridges with open forest canopy (Figure 3, Jucker, Hardwick, et al., 2018). In addition, plant size can be negatively related to stand density across topographic gradients, from valleys with low stem density and bigger trees (in height and diameter) to ridges with high stem density and smaller trees (Werner & Homeier, 2015). The high humidity and low temperature in valley can protect plant species during drought periods. However, the opening of forest canopies during droughts due to leaf loss can affect species adapted to low temperatures and high air humidity (Smith et al., 2019). The high transpiration rates are strongly related to high air moisture in the understory layer (Hardwick et al., 2015). In addition, ridges can show different microclimate conditions depending on the topographic aspect. For example, west‐facing ridges are warmer due to higher exposure to afternoon sun than east‐facing ridges that are exposed to morning sun (Stephenson, 1990). Therefore, microclimate variables, controlled by topography and vegetation structure, are strongly related to plant growth and mortality, especially for understory species.

## 6 BIOTIC FACTORS

### 6.1 Drought resilience of dominant and low abundance species

Plant tropical communities are complex systems due to high diversity and large differences in species abundances. The most basic classification of species in a plant community is based on abundance patterns and it separates species into dominants, subordinates, and transients (Grime, 1998; Whittaker, 1965). In a plant community, few species are classified as dominant species, which are the most abundant, and account for a higher proportion of the overall biomass of the community. On the other hand, low abundance species (subordinates and transients) represent lower amount of biomass, but are the main determinants of plant diversity (Grime, 1998; Mariotte, 2014; Whittaker, 1965). Dominant and low abundance species differ in their functional traits and play different roles in the ecosystem (Mariotte, 2014). In general, dominant species are competitively superior (Mariotte, 2014) and respond to environmental filtering, while low abundance species respond to niche differentiation (Maire et al., 2012). Low abundance species can promote the diversity of climbing plants (Garbin et al., 2012) and affect ecosystem functioning (Mariotte et al., 2015). Dominant species play an important role in structuring the species distributions (Wei et al., 2020) due to its homogenous pattern (Mariotte, 2014). For example, in a topographic gradient, they can occur in valleys and ridges (Hollunder et al., 2014). On the other hand, as low abundance species have a more aggregated spatial distribution (Garbin et al., 2016; Mariotte, 2014), it is expected that they occur in specific habitats and not across a whole topographic gradient (Hollunder et al., 2014). The response to drought depends on species and their spatial distribution and thus, taking into account topographical gradients (valleys, slopes, and ridges) and species groups (dominant vs. low abundance species) can improve our understanding of the processes that drive the response of tropical forests to severe droughts.

Low abundance species are less numerous, making populations of these species more vulnerable to local extinction induced by climate change (Greenwood et al., 2017). Furthermore, populations of low abundance species that occur with few individuals and with an aggregated spatial pattern (Mariotte, 2014) can be even more reduced due to habitat loss. On the other hand, dominant species are expected to be more resistant to local extinction due to their higher number of individuals (Greenwood et al., 2017). Therefore, locally, and perhaps even globally, species diversity can decrease under the future climate scenarios due to the role of low abundance species as main determinants of plant diversity. Dominant species play important roles on ecosystem functioning because of their large amount of biomass (Grime, 1998) and thus, they can act as a biotic filter in the establishment and survival of low abundance species (Khalil et al., 2019). The mortality of dominant species during droughts can also affect the performance of low abundance species. Furthermore, different species groups have distinct roles in the ecosystem during droughts. For instance, low abundance species modulate the quality of leaf litter, thus increasing the resistance in tropical forests by providing nutrients to other species (Hollunder et al., 2021).

### 6.2 Use of traits to unravel drought resilience mechanisms across topographical gradients

A functional trait is any feature which impacts fitness indirectly via effects on growth, reproduction, and survival at individual and species level (Diaz & Cabido, 2001; Violle et al., 2007). Traits can be measured at different levels, from tissue (e.g., stomatal and vein density), organ (e.g., specific leaf area [SLA], leaf dry matter content [LDMC], and woody density [WD]) to whole‐plant level (e.g., height and root‐to‐shoot ratio) (Pérez‐Harguindeguy et al., 2013). Trait combinations have the potential to explain the growth–mortality and resistance–recovery trade‐offs. Acquisitive traits linked to resource capture (e.g., leaf area), photosynthetic capacity (e.g., specific leaf area and leaf nitrogen content), and nutrient and water uptake (e.g., root length and diameter) generally have positive relationships with growth and negative relationships with mortality (Pérez‐Harguindeguy et al., 2013). Those traits are expected to be found in species from valleys with more water availability and nutrients (Apaza‐Quevedo et al., 2015). Thus, valleys are marked by more embolism‐vulnerable species (Oliveira et al., 2019). However, the widespread use of SLA to predict species drought resistance is likely to lead to misleading results (Delzon, 2015). In turn, conservative traits related to structural safety (e.g., wood density) or longevity (e.g., leaf dry matter content and leaf thickness) have negative relationships with growth, but are often positively related to survival (Poorter & Bongers, 2006). Those traits are expected to be found in species from slopes and ridges with less water availability and nutrients (Apaza‐Quevedo et al., 2015). Although acquisitive traits are strongly correlated with drought vulnerability, it is not yet clear whether it is a universal pattern. The vulnerability of plants from valley and wet forests depends not only on their traits but also on the interaction with environmental conditions and how drought can modify these conditions (Oliveira et al., 2019). For instance, plants in valleys and ridges are under different environmental conditions and thus, the drought resilience vary across topographical gradients (Hollunder et al., 2021; Zuleta et al., 2017). The mortality in valleys can be high when this habitat does not provide a shelter in terms of microclimate and resources to protect drought‐sensitive species. Severe droughts have a higher impact on dry habitats than valleys because those habitats are already under a climatic limit (Allen et al., 2017). The high mortality in ridges and slopes might indicate that conservative traits, known to increase drought resistance (Ceballos et al., 2021; Kraft et al., 2010; Shen et al., 2019), do not increase species resistance under a warmer and drier climate. Deciduousness species, usually from ridges and slopes, do not stabilize stem water potential during the drought season, which increases the risk of death by hydraulic failure (Souza et al., 2020).

Despite trees growing in valleys are less resistant against embolism (Garcia et al., 2022), valleys are expected to present less tree mortality during droughts (Hollunder et al., 2021; Nakagawa et al., 2000; Potts, 2003) and those habitats are marked by species presenting acquisitive traits (Apaza‐Quevedo et al., 2015). Thus, this might indicate that acquisitive traits do not make plant species more vulnerable to extreme droughts in valleys because such habitats provide resource and a suitable microclimate protecting drought‐sensitive species (Hollunder et al., 2021). In fact, when the topography is taken into account, acquisitive traits can be associated with high survival rates in valleys (Anderegg et al., 2019; Schwartz et al., 2020). In addition, despite species with low wood density have higher risk of xylem cavitation (Hoffmann et al., 2011), they can exhibit higher strength at lower construction costs (Larjavaara & Muller‐landau, 2010) and higher resistance to leaf desiccation than species with high wood density (Hoffmann et al., 2011). This might explain why species in valleys and moist forests present higher survival rates during extreme droughts than species from ridges and dry forests. However, more studies are necessary to test how traits modulate tree mortality across topographical habitats in tropical forests.

Hydraulic failure and carbon starvation have been widely debated in the literature to find mechanisms relying on drought‐induced tree mortality. Hydraulic failure occurs when the water transport to the canopy is reduced, resulting in desiccation and death of plant tissues (Hoffmann et al., 2011). Carbon starvation usually occurs, after the hydraulic failure, when drought duration is long enough to reduce storage of carbon reserves for maintenance of metabolism (McDowell et al., 2008). These mechanisms can be assessed by using different traits, which are classified according to their responses to different factors (Li et al., 2015): leaf economic traits (e.g., SLA and LDMC) and hydraulic traits (e.g., wood density and xylem vulnerability). In fact, wood density and specific leaf area are the main traits related to tree mortality, especially during severe droughts with negative SPEI values (Greenwood et al., 2017). Leaf economic traits are related to light use and carbon sequestration while hydraulic traits are related to gas exchange and water transport capacity (Li et al., 2015). It is expected that hydraulic traits are more associated with drought effects because water availability is the limiting factor during such periods (Brodribb, 2020). Indeed, hydraulic failure is the main mechanism explaining tree mortality during extreme droughts (Anderegg, Flint, et al., 2015; Anderegg et al., 2016; Powers et al., 2020) and forests with higher diversity in hydraulic traits are more resistant to drought impacts (Anderegg et al., 2018). Thus, water stress makes plants close their stomata to minimize water loss, which could lead to hydraulic failure and carbon starvation (Grossiord et al., 2020). Therefore, timing (resistance or recovery phase) should be taken into account when using functional traits as proxies to understand the causes of tree mortality.

A hydraulic perspective has attracted attention to predict tree mortality because plants can reduce water loss by shedding their leaves or closing their stomata to maintain plant water potential and avoid or reduce the risk of xylem cavitation (Vitória et al., 2019). Larger trees exhibit lower embolism resistance (Garcia et al., 2021) and they are more sensitive to hydraulic failure due to their higher demands for water comparing to smaller trees, which are expected to be more resistant (Moser et al., 2014; Phillips et al., 2010). In addition, understory plants are able to adjust their hydraulic systems in response to a dry period and changes in light availability (Giles et al., 2021). Nevertheless, small trees can face more water limitation due to their shallower roots (Gibbons & Newbery, 2002) and thus they also show higher mortality rates (Rocha et al., 2020). Hydraulic traits have been documented as important to predict drought‐induced tree mortality, such as: hydraulic safety margin (Anderegg et al., 2016; Powers et al., 2020), xylem vulnerability to embolism (Anderegg, Flint, et al., 2015; Brodribb, 2017; Liang et al., 2020; Oliveira et al., 2019), leaf water potential turgor loss point (Maréchaux et al., 2015), and woody density (Greenwood et al., 2017; Liang et al., 2020). In addition, our understanding about the mortality risk caused by hydraulic failure can be improved by combining stomatal control information with traits linked to xylem (Brodribb, 2017). Therefore, the use of hydraulic traits and leaf economic traits, as well as their relationships, can help understanding the cause of mortality and reduced growth. Focusing on traits related to water use and xylem vulnerability is a way to fill most of the remaining mechanistic gaps regarding drought effects (Delzon, 2015; Martinez‐Vilalta et al., 2019; Powers et al., 2020). Thus, ecologists have to include key traits directly and mechanistically relevant to plant survival and growth.

## 7 CONCLUSION

This literature review summarizes the role of abiotic and biotic factors mediating drought resilience in tropical forests. Our synthesis highlights that the regional climate conditions shape the forest types, and the topography controls biotic and abiotic factors at a local scale in moist and dry forests. Both dry tropical forests and ridges are more sensitive to droughts than moist tropical forest and valleys but the mechanisms explaining these patterns remain unknown. Field studies are essential to identify local and regional differences in drought resilience and to predict the future of tropical forests. This literature review also highlights the main gaps in drought resilience research, which are to: (1) identify mechanisms explaining both the growth–mortality and resistance–recovery trade‐offs; (2) understand how different functional groups (dominant vs. subordinate species, shade species vs. sun species, trees vs. shrubs) deal with droughts; (3) describe the physiological mechanisms explaining the forest resilience of different habitats (valley, slope, and ridge), forest types (moist vs. dry forests), and successional stages (secondary vs. primary forests); and (4) to understand how droughts change the microclimate in different habitats.

## AUTHOR CONTRIBUTIONS

**Renan Köpp Hollunder:** Conceptualization (lead); Investigation (lead); Project administration (lead); Writing – original draft (lead); Writing – review & editing (equal). **Mário Luís Garbin:** Funding acquisition (equal); Supervision (supporting); Writing – review & editing (equal). **Fabio Rubio Scarano:** Funding acquisition (equal); Supervision (supporting); Writing – review & editing (equal). **Pierre Mariotte:** Supervision (lead); Writing – review & editing (equal).

## CONFLICT OF INTEREST

The authors declare that they have no conflict of interest.

## Data Availability

There are no data available for this literature review.
